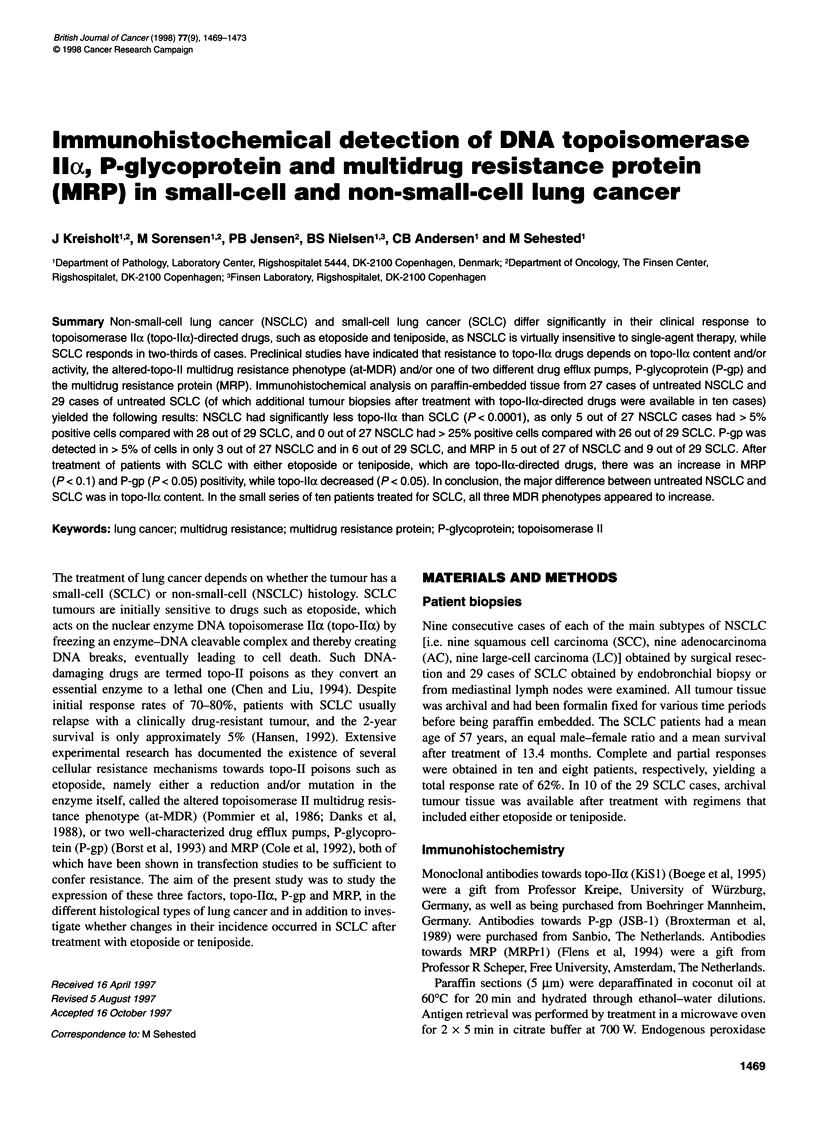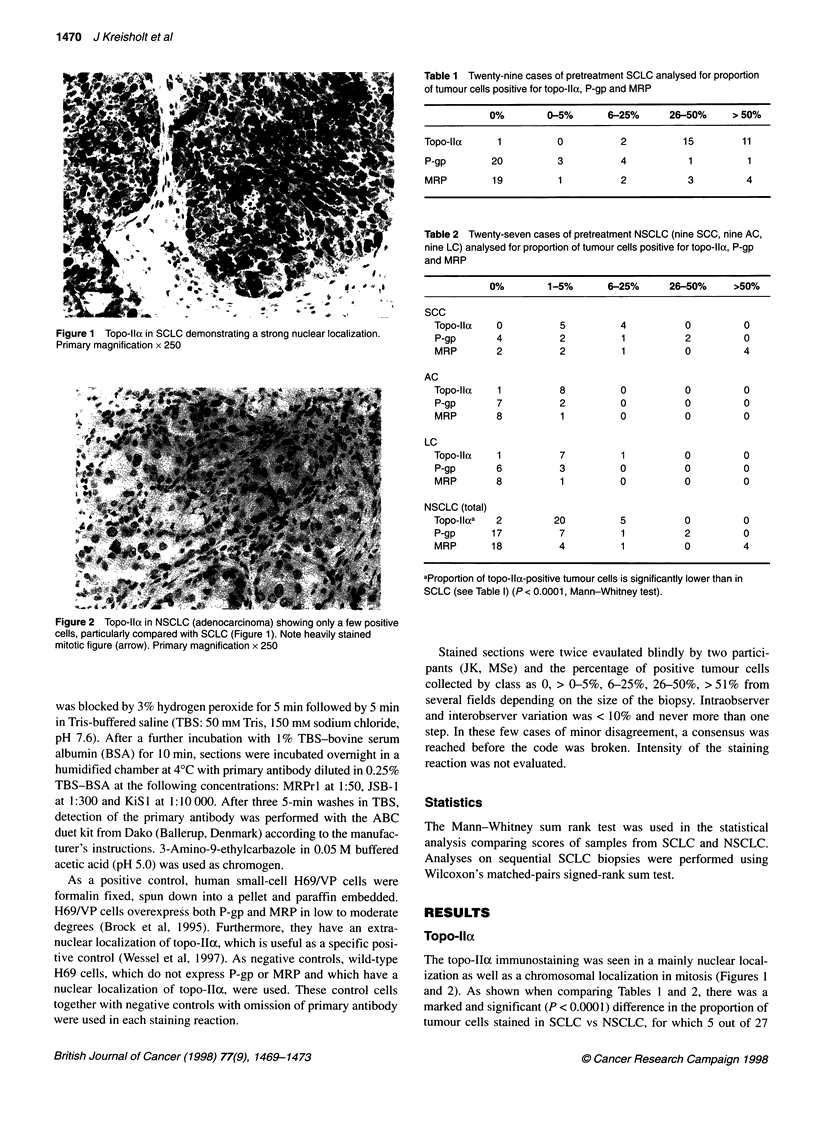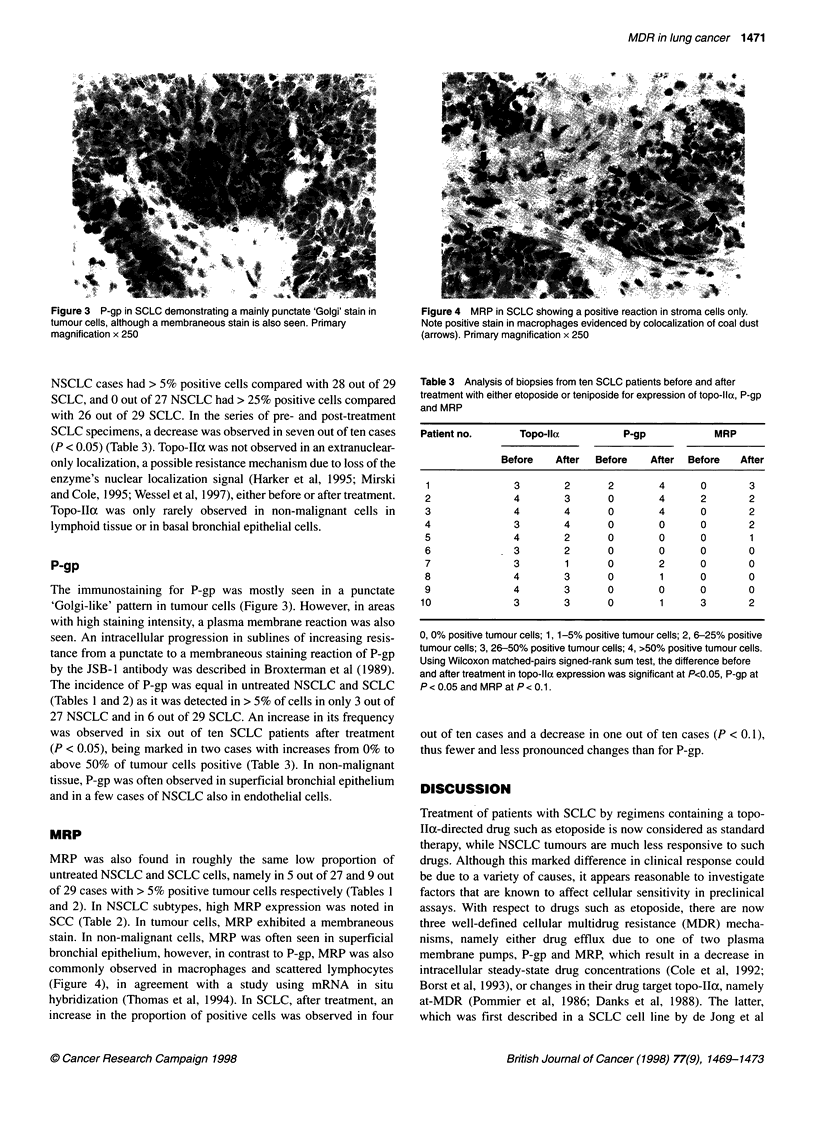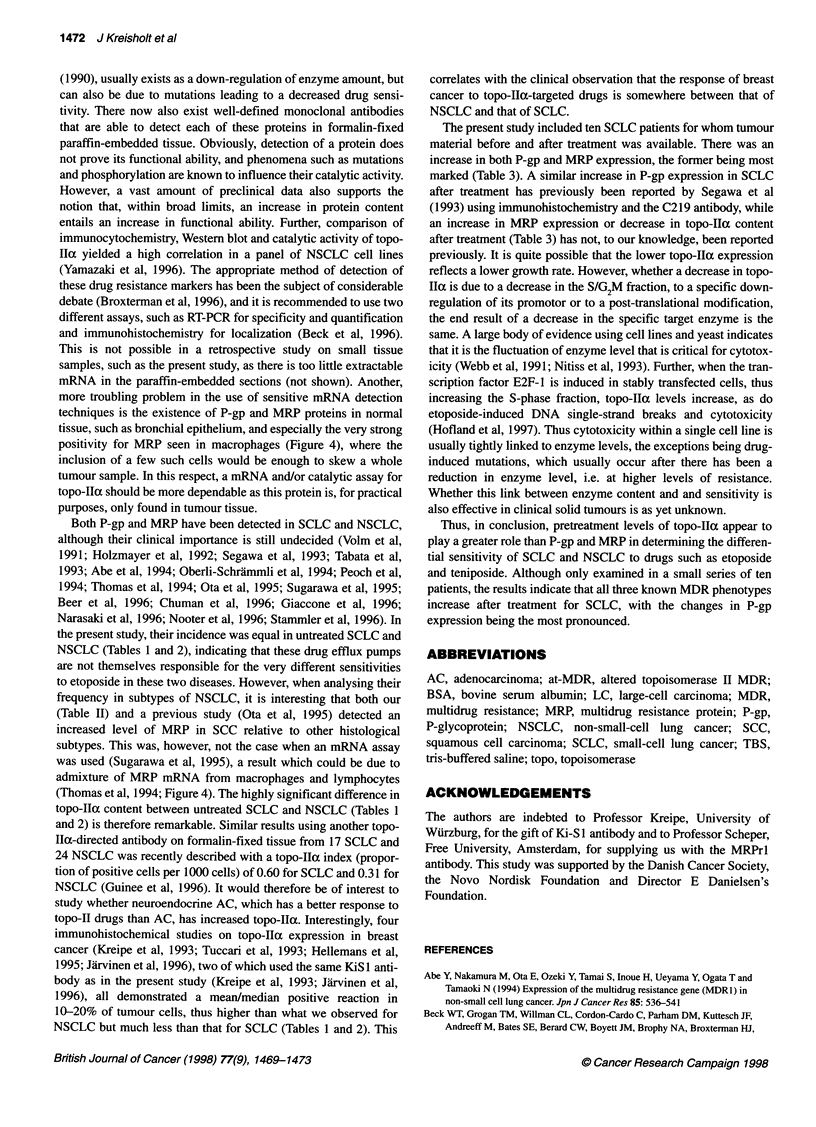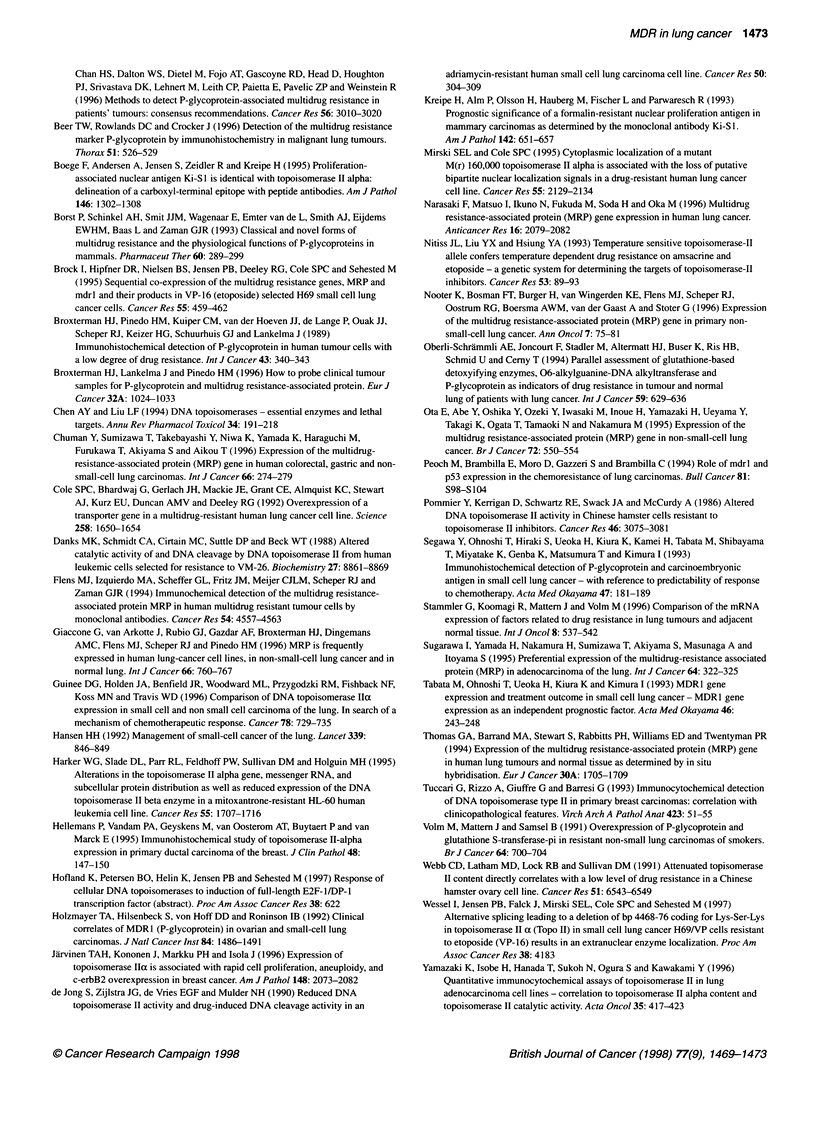# Immunohistochemical detection of DNA topoisomerase IIalpha, P-glycoprotein and multidrug resistance protein (MRP) in small-cell and non-small-cell lung cancer.

**DOI:** 10.1038/bjc.1998.241

**Published:** 1998-05

**Authors:** J. Kreisholt, M. Sorensen, P. B. Jensen, B. S. Nielsen, C. B. Andersen, M. Sehested

**Affiliations:** Department of Pathology, Laboratory Center, Rigshospitalet, Copenhagen, Denmark.

## Abstract

**Images:**


British Journal of Cancer (1998) 77(9), 1469–1473
© 1998 Cancer Research Campaign

### Immunohistochemical detection of DNA topoisomerase IIα, P-glycoprotein and multidrug resistance protein (MRP) in small-cell and non-small-cell lung cancer

**J Kreisholt[1,2], M Sorensen[1,2], PB Jensen[2], BS Nielsen[1,3], CB Andersen[1] and M Sehested[1]**

[1]Department of Pathology, Laboratory Center, Rigshospitalet 5444, DK-2100 Copenhagen, Denmark; [2]Department of Oncology, The Finsen Center, Rigshospitalet, DK-2100 Copenhagen; [3]Finsen Laboratory, Rigshospitalet, DK-2100 Copenhagen

**Summary** Non-small-cell lung cancer (NSCLC) and small-cell lung cancer (SCLC) differ significantly in their clinical response to topoisomerase IIα (topo-IIα)-directed drugs, such as etoposide and teniposide, as NSCLC is virtually insensitive to single-agent therapy, while SCLC responds in two-thirds of cases. Preclinical studies have indicated that resistance to topo-IIα drugs depends on topo-IIα content and/or activity, the altered-topo-II multidrug resistance phenotype (at-MDR) and/or one of two different drug efflux pumps, P-glycoprotein (P-gp) and the multidrug resistance protein (MRP). Immunohistochemical analysis on paraffin-embedded tissue from 27 cases of untreated NSCLC and 29 cases of untreated SCLC (of which additional tumour biopsies after treatment with topo-IIα-directed drugs were available in ten cases) yielded the following results: NSCLC had significantly less topo-IIα than SCLC ($P < 0.0001$), as only 5 out of 27 NSCLC cases had > 5% positive cells compared with 28 out of 29 SCLC, and 0 out of 27 NSCLC had > 25% positive cells compared with 26 out of 29 SCLC. P-gp was detected in > 5% of cells in only 3 out of 27 NSCLC and in 6 out of 29 SCLC, and MRP in 5 out of 27 of NSCLC and 9 out of 29 SCLC. After treatment of patients with SCLC with either etoposide or teniposide, which are topo-IIα-directed drugs, there was an increase in MRP ($P < 0.1$) and P-gp ($P < 0.05$) positivity, while topo-IIα decreased ($P < 0.05$). In conclusion, the major difference between untreated NSCLC and SCLC was in topo-IIα content. In the small series of ten patients treated for SCLC, all three MDR phenotypes appeared to increase.

**Keywords:** lung cancer; multidrug resistance; multidrug resistance protein; P-glycoprotein; topoisomerase II

The treatment of lung cancer depends on whether the tumour has a small-cell (SCLC) or non-small-cell (NSCLC) histology. SCLC tumours are initially sensitive to drugs such as etoposide, which acts on the nuclear enzyme DNA topoisomerase IIα (topo-IIα) by freezing an enzyme–DNA cleavable complex and thereby creating DNA breaks, eventually leading to cell death. Such DNA-damaging drugs are termed topo-II poisons as they convert an essential enzyme to a lethal one (Chen and Liu, 1994). Despite initial response rates of 70–80%, patients with SCLC usually relapse with a clinically drug-resistant tumour, and the 2-year survival is only approximately 5% (Hansen, 1992). Extensive experimental research has documented the existence of several cellular resistance mechanisms towards topo-II poisons such as etoposide, namely either a reduction and/or mutation in the enzyme itself, called the altered topoisomerase II multidrug resistance phenotype (at-MDR) (Pommier et al, 1986; Danks et al, 1988), or two well-characterized drug efflux pumps, P-glycoprotein (P-gp) (Borst et al, 1993) and MRP (Cole et al, 1992), both of which have been shown in transfection studies to be sufficient to confer resistance. The aim of the present study was to study the expression of these three factors, topo-IIα, P-gp and MRP, in the different histological types of lung cancer and in addition to investigate whether changes in their incidence occurred in SCLC after treatment with etoposide or teniposide.

*Received 16 April 1997*
*Revised 5 August 1997*
*Accepted 16 October 1997*

*Correspondence to:* M Sehested

### MATERIALS AND METHODS

### Patient biopsies

Nine consecutive cases of each of the main subtypes of NSCLC [i.e. nine squamous cell carcinoma (SCC), nine adenocarcinoma (AC), nine large-cell carcinoma (LC)] obtained by surgical resection and 29 cases of SCLC obtained by endobronchial biopsy or from mediastinal lymph nodes were examined. All tumour tissue was archival and had been formalin fixed for various time periods before being paraffin embedded. The SCLC patients had a mean age of 57 years, an equal male–female ratio and a mean survival after treatment of 13.4 months. Complete and partial responses were obtained in ten and eight patients, respectively, yielding a total response rate of 62%. In 10 of the 29 SCLC cases, archival tumour tissue was available after treatment with regimens that included either etoposide or teniposide.

### Immunohistochemistry

Monoclonal antibodies towards topo-IIα (KiS1) (Boege et al, 1995) were a gift from Professor Kreipe, University of Würzburg, Germany, as well as being purchased from Boehringer Mannheim, Germany. Antibodies towards P-gp (JSB-1) (Broxterman et al, 1989) were purchased from Sanbio, The Netherlands. Antibodies towards MRP (MRPr1) (Flens et al, 1994) were a gift from Professor R Scheper, Free University, Amsterdam, The Netherlands.

Paraffin sections (5 μm) were deparaffinated in coconut oil at 60°C for 20 min and hydrated through ethanol–water dilutions. Antigen retrieval was performed by treatment in a microwave oven for 2 × 5 min in citrate buffer at 700 W. Endogenous peroxidase

Figure 1 Topo-IIα in SCLC demonstrating a strong nuclear localization. Primary magnification × 250

Figure 2 Topo-IIα in NSCLC (adenocarcinoma) showing only a few positive cells, particularly compared with SCLC (Figure 1). Note heavily stained mitotic figure (arrow). Primary magnification × 250

was blocked by 3% hydrogen peroxide for 5 min followed by 5 min in Tris-buffered saline (TBS: 50 mM Tris, 150 mM sodium chloride, pH 7.6). After a further incubation with 1% TBS–bovine serum albumin (BSA) for 10 min, sections were incubated overnight in a humidified chamber at 4°C with primary antibody diluted in 0.25% TBS–BSA at the following concentrations: MRPr1 at 1:50, JSB-1 at 1:300 and KiS1 at 1:10 000. After three 5-min washes in TBS, detection of the primary antibody was performed with the ABC duet kit from Dako (Ballerup, Denmark) according to the manufacturer's instructions. 3-Amino-9-ethylcarbazole in 0.05 M buffered acetic acid (pH 5.0) was used as chromogen.

As a positive control, human small-cell H69/VP cells were formalin fixed, spun down into a pellet and paraffin embedded. H69/VP cells overexpress both P-gp and MRP in low to moderate degrees (Brock et al, 1995). Furthermore, they have an extra-nuclear localization of topo-IIα, which is useful as a specific positive control (Wessel et al, 1997). As negative controls, wild-type H69 cells, which do not express P-gp or MRP and which have a nuclear localization of topo-IIα, were used. These control cells together with negative controls with omission of primary antibody were used in each staining reaction.

Table 1 Twenty-nine cases of pretreatment SCLC analysed for proportion of tumour cells positive for topo-IIα, P-gp and MRP

| | 0% | 0–5% | 6–25% | 26–50% | > 50% |
|---|---|---|---|---|---|
| Topo-IIα | 1 | 0 | 2 | 15 | 11 |
| P-gp | 20 | 3 | 4 | 1 | 1 |
| MRP | 19 | 1 | 2 | 3 | 4 |

Table 2 Twenty-seven cases of pretreatment NSCLC (nine SCC, nine AC, nine LC) analysed for proportion of tumour cells positive for topo-IIα, P-gp and MRP

| | 0% | 1–5% | 6–25% | 26–50% | >50% |
|---|---|---|---|---|---|
| SCC | | | | | |
| Topo-IIα | 0 | 5 | 4 | 0 | 0 |
| P-gp | 4 | 2 | 1 | 2 | 0 |
| MRP | 2 | 2 | 1 | 0 | 4 |
| AC | | | | | |
| Topo-IIα | 1 | 8 | 0 | 0 | 0 |
| P-gp | 7 | 2 | 0 | 0 | 0 |
| MRP | 8 | 1 | 0 | 0 | 0 |
| LC | | | | | |
| Topo-IIα | 1 | 7 | 1 | 0 | 0 |
| P-gp | 6 | 3 | 0 | 0 | 0 |
| MRP | 8 | 1 | 0 | 0 | 0 |
| NSCLC (total) | | | | | |
| Topo-IIα[a] | 2 | 20 | 5 | 0 | 0 |
| P-gp | 17 | 7 | 1 | 2 | 0 |
| MRP | 18 | 4 | 1 | 0 | 4 |

[a]Proportion of topo-IIα-positive tumour cells is significantly lower than in SCLC (see Table I) ($P < 0.0001$, Mann–Whitney test).

Stained sections were twice evaulated blindly by two participants (JK, MSe) and the percentage of positive tumour cells collected by class as 0, > 0–5%, 6–25%, 26–50%, > 51% from several fields depending on the size of the biopsy. Intraobserver and interobserver variation was < 10% and never more than one step. In these few cases of minor disagreement, a consensus was reached before the code was broken. Intensity of the staining reaction was not evaluated.

### Statistics

The Mann–Whitney sum rank test was used in the statistical analysis comparing scores of samples from SCLC and NSCLC. Analyses on sequential SCLC biopsies were performed using Wilcoxon's matched-pairs signed-rank sum test.

### RESULTS

### Topo-IIα

The topo-IIα immunostaining was seen in a mainly nuclear localization as well as a chromosomal localization in mitosis (Figures 1 and 2). As shown when comparing Tables 1 and 2, there was a marked and significant ($P < 0.0001$) difference in the proportion of tumour cells stained in SCLC vs NSCLC, for which 5 out of 27

© Cancer Research Campaign 1998

**Figure 3** P-gp in SCLC demonstrating a mainly punctate 'Golgi' stain in tumour cells, although a membraneous stain is also seen. Primary magnification × 250

**Figure 4** MRP in SCLC showing a positive reaction in stroma cells only. Note positive stain in macrophages evidenced by colocalization of coal dust (arrows). Primary magnification × 250

NSCLC cases had > 5% positive cells compared with 28 out of 29 SCLC, and 0 out of 27 NSCLC had > 25% positive cells compared with 26 out of 29 SCLC. In the series of pre- and post-treatment SCLC specimens, a decrease was observed in seven out of ten cases ($P < 0.05$) (Table 3). Topo-IIα was not observed in an extranuclear-only localization, a possible resistance mechanism due to loss of the enzyme's nuclear localization signal (Harker et al, 1995; Mirski and Cole, 1995; Wessel et al, 1997), either before or after treatment. Topo-IIα was only rarely observed in non-malignant cells in lymphoid tissue or in basal bronchial epithelial cells.

### P-gp

The immunostaining for P-gp was mostly seen in a punctate 'Golgi-like' pattern in tumour cells (Figure 3). However, in areas with high staining intensity, a plasma membrane reaction was also seen. An intracellular progression in sublines of increasing resistance from a punctate to a membraneous staining reaction of P-gp by the JSB-1 antibody was described in Broxterman et al (1989). The incidence of P-gp was equal in untreated NSCLC and SCLC (Tables 1 and 2) as it was detected in > 5% of cells in only 3 out of 27 NSCLC and in 6 out of 29 SCLC. An increase in its frequency was observed in six out of ten SCLC patients after treatment ($P < 0.05$), being marked in two cases with increases from 0% to above 50% of tumour cells positive (Table 3). In non-malignant tissue, P-gp was often observed in superficial bronchial epithelium and in a few cases of NSCLC also in endothelial cells.

### MRP

MRP was also found in roughly the same low proportion of untreated NSCLC and SCLC cells, namely in 5 out of 27 and 9 out of 29 cases with > 5% positive tumour cells respectively (Tables 1 and 2). In NSCLC subtypes, high MRP expression was noted in SCC (Table 2). In tumour cells, MRP exhibited a membraneous stain. In non-malignant cells, MRP was often seen in superficial bronchial epithelium, however, in contrast to P-gp, MRP was also commonly observed in macrophages and scattered lymphocytes (Figure 4), in agreement with a study using mRNA in situ hybridization (Thomas et al, 1994). In SCLC, after treatment, an increase in the proportion of positive cells was observed in four out of ten cases and a decrease in one out of ten cases ($P < 0.1$), thus fewer and less pronounced changes than for P-gp.

**Table 3** Analysis of biopsies from ten SCLC patients before and after treatment with either etoposide or teniposide for expression of topo-IIα, P-gp and MRP

| Patient no. | Topo-IIα | | P-gp | | MRP | |
|---|---|---|---|---|---|---|
| | Before | After | Before | After | Before | After |
| 1 | 3 | 2 | 2 | 4 | 0 | 3 |
| 2 | 4 | 3 | 0 | 4 | 2 | 2 |
| 3 | 4 | 4 | 0 | 4 | 0 | 2 |
| 4 | 3 | 4 | 0 | 0 | 0 | 2 |
| 5 | 4 | 2 | 0 | 0 | 0 | 1 |
| 6 | 3 | 2 | 0 | 0 | 0 | 0 |
| 7 | 3 | 1 | 0 | 2 | 0 | 0 |
| 8 | 4 | 3 | 0 | 1 | 0 | 0 |
| 9 | 4 | 3 | 0 | 0 | 0 | 0 |
| 10 | 3 | 3 | 0 | 1 | 3 | 2 |

0, 0% positive tumour cells; 1, 1–5% positive tumour cells; 2, 6–25% positive tumour cells; 3, 26–50% positive tumour cells; 4, >50% positive tumour cells. Using Wilcoxon matched-pairs signed-rank sum test, the difference before and after treatment in topo-IIα expression was significant at $P<0.05$, P-gp at $P < 0.05$ and MRP at $P < 0.1$.

### DISCUSSION

Treatment of patients with SCLC by regimens containing a topo-IIα-directed drug such as etoposide is now considered as standard therapy, while NSCLC tumours are much less responsive to such drugs. Although this marked difference in clinical response could be due to a variety of causes, it appears reasonable to investigate factors that are known to affect cellular sensitivity in preclinical assays. With respect to drugs such as etoposide, there are now three well-defined cellular multidrug resistance (MDR) mechanisms, namely either drug efflux due to one of two plasma membrane pumps, P-gp and MRP, which result in a decrease in intracellular steady-state drug concentrations (Cole et al, 1992; Borst et al, 1993), or changes in their drug target topo-IIα, namely at-MDR (Pommier et al, 1986; Danks et al, 1988). The latter, which was first described in a SCLC cell line by de Jong et al

© Cancer Research Campaign 1998

(1990), usually exists as a down-regulation of enzyme amount, but can also be due to mutations leading to a decreased drug sensitivity. There now also exist well-defined monoclonal antibodies that are able to detect each of these proteins in formalin-fixed paraffin-embedded tissue. Obviously, detection of a protein does not prove its functional ability, and phenomena such as mutations and phosphorylation are known to influence their catalytic activity. However, a vast amount of preclinical data also supports the notion that, within broad limits, an increase in protein content entails an increase in functional ability. Further, comparison of immunocytochemistry, Western blot and catalytic activity of topo-IIα yielded a high correlation in a panel of NSCLC cell lines (Yamazaki et al, 1996). The appropriate method of detection of these drug resistance markers has been the subject of considerable debate (Broxterman et al, 1996), and it is recommended to use two different assays, such as RT-PCR for specificity and quantification and immunohistochemistry for localization (Beck et al, 1996). This is not possible in a retrospective study on small tissue samples, such as the present study, as there is too little extractable mRNA in the paraffin-embedded sections (not shown). Another, more troubling problem in the use of sensitive mRNA detection techniques is the existence of P-gp and MRP proteins in normal tissue, such as bronchial epithelium, and especially the very strong positivity for MRP seen in macrophages (Figure 4), where the inclusion of a few such cells would be enough to skew a whole tumour sample. In this respect, a mRNA and/or catalytic assay for topo-IIα should be more dependable as this protein is, for practical purposes, only found in tumour tissue.

Both P-gp and MRP have been detected in SCLC and NSCLC, although their clinical importance is still undecided (Volm et al, 1991; Holzmayer et al, 1992; Segawa et al, 1993; Tabata et al, 1993; Abe et al, 1994; Oberli-Schrämmli et al, 1994; Peoch et al, 1994; Thomas et al, 1994; Ota et al, 1995; Sugarawa et al, 1995; Beer et al, 1996; Chuman et al, 1996; Giaccone et al, 1996; Narasaki et al, 1996; Nooter et al, 1996; Stammler et al, 1996). In the present study, their incidence was equal in untreated SCLC and NSCLC (Tables 1 and 2), indicating that these drug efflux pumps are not themselves responsible for the very different sensitivities to etoposide in these two diseases. However, when analysing their frequency in subtypes of NSCLC, it is interesting that both our (Table II) and a previous study (Ota et al, 1995) detected an increased level of MRP in SCC relative to other histological subtypes. This was, however, not the case when an mRNA assay was used (Sugarawa et al, 1995), a result which could be due to admixture of MRP mRNA from macrophages and lymphocytes (Thomas et al, 1994; Figure 4). The highly significant difference in topo-IIα content between untreated SCLC and NSCLC (Tables 1 and 2) is therefore remarkable. Similar results using another topo-IIα-directed antibody on formalin-fixed tissue from 17 SCLC and 24 NSCLC was recently described with a topo-IIα index (proportion of positive cells per 1000 cells) of 0.60 for SCLC and 0.31 for NSCLC (Guinee et al, 1996). It would therefore be of interest to study whether neuroendocrine AC, which has a better response to topo-II drugs than AC, has increased topo-IIα. Interestingly, four immunohistochemical studies on topo-IIα expression in breast cancer (Kreipe et al, 1993; Tuccari et al, 1993; Hellemans et al, 1995; Järvinen et al, 1996), two of which used the same KiS1 antibody as in the present study (Kreipe et al, 1993; Järvinen et al, 1996), all demonstrated a mean/median positive reaction in 10–20% of tumour cells, thus higher than what we observed for NSCLC but much less than that for SCLC (Tables 1 and 2). This correlates with the clinical observation that the response of breast cancer to topo-IIα-targeted drugs is somewhere between that of NSCLC and that of SCLC.

The present study included ten SCLC patients for whom tumour material before and after treatment was available. There was an increase in both P-gp and MRP expression, the former being most marked (Table 3). A similar increase in P-gp expression in SCLC after treatment has previously been reported by Segawa et al (1993) using immunohistochemistry and the C219 antibody, while an increase in MRP expression or decrease in topo-IIα content after treatment (Table 3) has not, to our knowledge, been reported previously. It is quite possible that the lower topo-IIα expression reflects a lower growth rate. However, whether a decrease in topo-IIα is due to a decrease in the $S/G_2M$ fraction, to a specific down-regulation of its promotor or to a post-translational modification, the end result of a decrease in the specific target enzyme is the same. A large body of evidence using cell lines and yeast indicates that it is the fluctuation of enzyme level that is critical for cytotoxicity (Webb et al, 1991; Nitiss et al, 1993). Further, when the transcription factor E2F-1 is induced in stably transfected cells, thus increasing the S-phase fraction, topo-IIα levels increase, as do etoposide-induced DNA single-strand breaks and cytotoxicity (Hofland et al, 1997). Thus cytotoxicity within a single cell line is usually tightly linked to enzyme levels, the exceptions being drug-induced mutations, which usually occur after there has been a reduction in enzyme level, i.e. at higher levels of resistance. Whether this link between enzyme content and and sensitivity is also effective in clinical solid tumours is as yet unknown.

Thus, in conclusion, pretreatment levels of topo-IIα appear to play a greater role than P-gp and MRP in determining the differential sensitivity of SCLC and NSCLC to drugs such as etoposide and teniposide. Although only examined in a small series of ten patients, the results indicate that all three known MDR phenotypes increase after treatment for SCLC, with the changes in P-gp expression being the most pronounced.

### ABBREVIATIONS

AC, adenocarcinoma; at-MDR, altered topoisomerase II MDR; BSA, bovine serum albumin; LC, large-cell carcinoma; MDR, multidrug resistance; MRP, multidrug resistance protein; P-gp, P-glycoprotein; NSCLC, non-small-cell lung cancer; SCC, squamous cell carcinoma; SCLC, small-cell lung cancer; TBS, tris-buffered saline; topo, topoisomerase

### ACKNOWLEDGEMENTS

The authors are indebted to Professor Kreipe, University of Würzburg, for the gift of Ki-S1 antibody and to Professor Scheper, Free University, Amsterdam, for supplying us with the MRPr1 antibody. This study was supported by the Danish Cancer Society, the Novo Nordisk Foundation and Director E Danielsen's Foundation.

### REFERENCES

Abe Y, Nakamura M, Ota E, Ozeki Y, Tamai S, Inoue H, Ueyama Y, Ogata T and Tamaoki N (1994) Expression of the multidrug resistance gene (MDR1) in non-small cell lung cancer. *Jpn J Cancer Res* **85**: 536–541

Beck WT, Grogan TM, Willman CL, Cordon-Cardo C, Parham DM, Kuttesch JF, Andreeff M, Bates SE, Berard CW, Boyett JM, Brophy NA, Broxterman HJ,

© Cancer Research Campaign 1998

Chan HS, Dalton WS, Dietel M, Fojo AT, Gascoyne RD, Head D, Houghton PJ, Srivastava DK, Lehnert M, Leith CP, Paietta E, Pavelic ZP and Weinstein R (1996) Methods to detect P-glycoprotein-associated multidrug resistance in patients' tumours: consensus recommendations. *Cancer Res* **56**: 3010–3020

Beer TW, Rowlands DC and Crocker J (1996) Detection of the multidrug resistance marker P-glycoprotein by immunohistochemistry in malignant lung tumours. *Thorax* **51**: 526–529

Boege F, Andersen A, Jensen S, Zeidler R and Kreipe H (1995) Proliferation-associated nuclear antigen Ki-S1 is identical with topoisomerase II alpha: delineation of a carboxyl-terminal epitope with peptide antibodies. *Am J Pathol* **146**: 1302–1308

Borst P, Schinkel AH, Smit JJM, Wagenaar E, Emter van de L, Smith AJ, Eijdems EWHM, Baas L and Zaman GJR (1993) Classical and novel forms of multidrug resistance and the physiological functions of P-glycoproteins in mammals. *Pharmaceut Ther* **60**: 289–299

Brock I, Hipfner DR, Nielsen BS, Jensen PB, Deeley RG, Cole SPC and Sehested M (1995) Sequential co-expression of the multidrug resistance genes, MRP and mdr1 and their products in VP-16 (etoposide) selected H69 small cell lung cancer cells. *Cancer Res* **55**: 459–462

Broxterman HJ, Pinedo HM, Kuiper CM, van der Hoeven JJ, de Lange P, Ouak JJ, Scheper RJ, Keizer HG, Schuurhuis GJ and Lankelma J (1989) Immunohistochemical detection of P-glycoprotein in human tumour cells with a low degree of drug resistance. *Int J Cancer* **43**: 340–343

Broxterman HJ, Lankelma J and Pinedo HM (1996) How to probe clinical tumour samples for P-glycoprotein and multidrug resistance-associated protein. *Eur J Cancer* **32A**: 1024–1033

Chen AY and Liu LF (1994) DNA topoisomerases – essential enzymes and lethal targets. *Annu Rev Pharmacol Toxicol* **34**: 191–218

Chuman Y, Sumizawa T, Takebayashi Y, Niwa K, Yamada K, Haraguchi M, Furukawa T, Akiyama S and Aikou T (1996) Expression of the multidrug-resistance-associated protein (MRP) gene in human colorectal, gastric and non-small-cell lung carcinomas. *Int J Cancer* **66**: 274–279

Cole SPC, Bhardwaj G, Gerlach JH, Mackie JE, Grant CE, Almquist KC, Stewart AJ, Kurz EU, Duncan AMV and Deeley RG (1992) Overexpression of a transporter gene in a multidrug-resistant human lung cancer cell line. *Science* **258**: 1650–1654

Danks MK, Schmidt CA, Cirtain MC, Suttle DP and Beck WT (1988) Altered catalytic activity of and DNA cleavage by DNA topoisomerase II from human leukemic cells selected for resistance to VM-26. *Biochemistry* **27**: 8861–8869

Flens MJ, Izquierdo MA, Scheffer GL, Fritz JM, Meijer CJLM, Scheper RJ and Zaman GJR (1994) Immunochemical detection of the multidrug resistance-associated protein MRP in human multidrug resistant tumour cells by monoclonal antibodies. *Cancer Res* **54**: 4557–4563

Giaccone G, van Arkotte J, Rubio GJ, Gazdar AF, Broxterman HJ, Dingemans AMC, Flens MJ, Scheper RJ and Pinedo HM (1996) MRP is frequently expressed in human lung-cancer cell lines, in non-small-cell lung cancer and in normal lung. *Int J Cancer* **66**: 760–767

Guinee DG, Holden JA, Benfield JR, Woodward ML, Przygodzki RM, Fishback NF, Koss MN and Travis WD (1996) Comparison of DNA topoisomerase IIα expression in small cell and non small cell carcinoma of the lung. In search of a mechanism of chemotherapeutic response. *Cancer* **78**: 729–735

Hansen HH (1992) Management of small-cell cancer of the lung. *Lancet* **339**: 846–849

Harker WG, Slade DL, Parr RL, Feldhoff PW, Sullivan DM and Holguin MH (1995) Alterations in the topoisomerase II alpha gene, messenger RNA, and subcellular protein distribution as well as reduced expression of the DNA topoisomerase II beta enzyme in a mitoxantrone-resistant HL-60 human leukemia cell line. *Cancer Res* **55**: 1707–1716

Hellemans P, Vandam PA, Geyskens M, van Oosterom AT, Buytaert P and van Marck E (1995) Immunohistochemical study of topoisomerase II-alpha expression in primary ductal carcinoma of the breast. *J Clin Pathol* **48**: 147–150

Hofland K, Petersen BO, Helin K, Jensen PB and Sehested M (1997) Response of cellular DNA topoisomerases to induction of full-length E2F-1/DP-1 transcription factor (abstract). *Proc Am Assoc Cancer Res* **38**: 622

Holzmayer TA, Hilsenbeck S, von Hoff DD and Roninson IB (1992) Clinical correlates of MDR1 (P-glycoprotein) in ovarian and small-cell lung carcinomas. *J Natl Cancer Inst* **84**: 1486–1491

Järvinen TAH, Kononen J, Markku PH and Isola J (1996) Expression of topoisomerase IIα is associated with rapid cell proliferation, aneuploidy, and c-erbB2 overexpression in breast cancer. *Am J Pathol* **148**: 2073–2082

de Jong S, Zijlstra JG, de Vries EGF and Mulder NH (1990) Reduced DNA topoisomerase II activity and drug-induced DNA cleavage activity in an adriamycin-resistant human small cell lung carcinoma cell line. *Cancer Res* **50**: 304–309

Kreipe H, Alm P, Olsson H, Hauberg M, Fischer L and Parwaresch R (1993) Prognostic significance of a formalin-resistant nuclear proliferation antigen in mammary carcinomas as determined by the monoclonal antibody Ki-S1. *Am J Pathol* **142**: 651–657

Mirski SEL and Cole SPC (1995) Cytoplasmic localization of a mutant M(r) 160,000 topoisomerase II alpha is associated with the loss of putative bipartite nuclear localization signals in a drug-resistant human lung cancer cell line. *Cancer Res* **55**: 2129–2134

Narasaki F, Matsuo I, Ikuno N, Fukuda M, Soda H and Oka M (1996) Multidrug resistance-associated protein (MRP) gene expression in human lung cancer. *Anticancer Res* **16**: 2079–2082

Nitiss JL, Liu YX and Hsiung YA (1993) Temperature sensitive topoisomerase-II allele confers temperature dependent drug resistance on amsacrine and etoposide – a genetic system for determining the targets of topoisomerase-II inhibitors. *Cancer Res* **53**: 89–93

Nooter K, Bosman FT, Burger H, van Wingerden KE, Flens MJ, Scheper RJ, Oostrum RG, Boersma AWM, van der Gaast A and Stoter G (1996) Expression of the multidrug resistance-associated protein (MRP) gene in primary non-small-cell lung cancer. *Ann Oncol* **7**: 75–81

Oberli-Schrämmli AE, Joncourt F, Stadler M, Altermatt HJ, Buser K, Ris HB, Schmid U and Cerny T (1994) Parallel assessment of glutathione-based detoxyifying enzymes, O6-alkylguanine-DNA alkyltransferase and P-glycoprotein as indicators of drug resistance in tumour and normal lung of patients with lung cancer. *Int J Cancer* **59**: 629–636

Ota E, Abe Y, Oshika Y, Ozeki Y, Iwasaki M, Inoue H, Yamazaki H, Ueyama Y, Takagi K, Ogata T, Tamaoki N and Nakamura M (1995) Expression of the multidrug resistance-associated protein (MRP) gene in non-small-cell lung cancer. *Br J Cancer* **72**: 550–554

Peoch M, Brambilla E, Moro D, Gazzeri S and Brambilla C (1994) Role of mdr1 and p53 expression in the chemoresistance of lung carcinomas. *Bull Cancer* **81**: S98–S104

Pommier Y, Kerrigan D, Schwartz RE, Swack JA and McCurdy A (1986) Altered DNA topoisomerase II activity in Chinese hamster cells resistant to topoisomerase II inhibitors. *Cancer Res* **46**: 3075–3081

Segawa Y, Ohnoshi T, Hiraki S, Ueoka H, Kiura K, Kamei H, Tabata M, Shibayama T, Miyatake K, Genba K, Matsumura T and Kimura I (1993) Immunohistochemical detection of P-glycoprotein and carcinoembryonic antigen in small cell lung cancer – with reference to predictability of response to chemotherapy. *Acta Med Okayama* **47**: 181–189

Stammler G, Koomagi R, Mattern J and Volm M (1996) Comparison of the mRNA expression of factors related to drug resistance in lung tumours and adjacent normal tissue. *Int J Oncol* **8**: 537–542

Sugarawa I, Yamada H, Nakamura H, Sumizawa T, Akiyama S, Masunaga A and Itoyama S (1995) Preferential expression of the multidrug-resistance associated protein (MRP) in adenocarcinoma of the lung. *Int J Cancer* **64**: 322–325

Tabata M, Ohnoshi T, Ueoka H, Kiura K and Kimura I (1993) MDR1 gene expression and treatment outcome in small cell lung cancer – MDR1 gene expression as an independent prognostic factor. *Acta Med Okayama* **46**: 243–248

Thomas GA, Barrand MA, Stewart S, Rabbitts PH, Williams ED and Twentyman PR (1994) Expression of the multidrug resistance-associated protein (MRP) gene in human lung tumours and normal tissue as determined by in situ hybridisation. *Eur J Cancer* **30A**: 1705–1709

Tuccari G, Rizzo A, Giuffre G and Barresi G (1993) Immunocytochemical detection of DNA topoisomerase type II in primary breast carcinomas: correlation with clinicopathological features. *Virch Arch A Pathol Anat* **423**: 51–55

Volm M, Mattern J and Samsel B (1991) Overexpression of P-glycoprotein and glutathione S-transferase-pi in resistant non-small lung carcinomas of smokers. *Br J Cancer* **64**: 700–704

Webb CD, Latham MD, Lock RB and Sullivan DM (1991) Attenuated topisomerase II content directly correlates with a low level of drug resistance in a Chinese hamster ovary cell line. *Cancer Res* **51**: 6543–6549

Wessel I, Jensen PB, Falck J, Mirski SEL, Cole SPC and Sehested M (1997) Alternative splicing leading to a deletion of bp 4468-76 coding for Lys-Ser-Lys in topoisomerase II α (Topo II) in small cell lung cancer H69/VP cells resistant to etoposide (VP-16) results in an extranuclear enzyme localization. *Proc Am Assoc Cancer Res* **38**: 4183

Yamazaki K, Isobe H, Hanada T, Sukoh N, Ogura S and Kawakami Y (1996) Quantitative immunocytochemical assays of topoisomerase II in lung adenocarcinoma cell lines – correlation to topoisomerase II alpha content and topoisomerase II catalytic activity. *Acta Oncol* **35**: 417–423

© Cancer Research Campaign 1998